# The Effects of Patient Positioning on the Outcome During Posterior Cranial Fossa and Pineal Region Surgery

**DOI:** 10.3389/fsurg.2020.00009

**Published:** 2020-03-13

**Authors:** Ana Mavarez-Martinez, Lusine A. Israelyan, Suren Soghomonyan, Juan Fiorda-Diaz, Gurneet Sandhu, Vadim N. Shimansky, Mario Ammirati, Marilly Palettas, Andrei Yu Lubnin, Sergio D. Bergese

**Affiliations:** ^1^Department of Anesthesiology, Stony Brook Medicine, Stony Brook, NY, United States; ^2^Department of Anesthesiology, Burdenko Neurosurgical Institute, Moscow, Russia; ^3^Department of Anesthesiology, The Ohio State University Wexner Medical Center, Columbus, OH, United States; ^4^Department of Posterior Cranial Fossa and Scull Base Surgery, Burdenko Neurosurgical Institute, Moscow, Russia; ^5^Department of Neurological Surgery, Mercy Health St. Rita Medical Center, Lima, OH, United States; ^6^Department of Biology, College of Science and Technology, Sbarro Health Organization, Temple University, Philadelphia, PA, United States; ^7^Center for Biostatistics, The Ohio State University, Columbus, OH, United States

**Keywords:** sitting position, horizontal position, craniotomy, posterior fossa, pineal region

## Abstract

**Background:** Surgery on posterior cranial fossa (PCF) and pineal region (PR) carries the risks of intraoperative trauma to the brainstem structures, blood loss, venous air embolism (VAE), cardiovascular instability, and other complications. Success in surgery, among other factors, depends on selecting the optimal patient position. Our objective was to find associations between patient positioning, incidence of intraoperative complications, neurological recovery, and the extent of surgery.

**Methods:** This observational study was conducted in two medical centers: The Ohio State University Wexner Medical Center (USA) and The Burdenko Neurosurgical Institute (Russian Federation). Patients were distributed in two groups based on the surgical position: sitting position (SP) or horizontal position (HP). The inclusion criteria were adult patients with space-occupying or vascular lesions requiring an open PCF or PR surgery. Perioperative variables were recorded and summarized using descriptive statistics. The post-treatment survival, functional outcome, and patient satisfaction were assessed at 3 months.

**Results:** A total of 109 patients were included in the study: 53 in SP and 56 in HP. A higher proportion of patients in the HP patients had >300 mL intraoperative blood loss compared to the SP group (32 vs. 13%; *p* = 0.0250). Intraoperative VAE was diagnosed in 40% of SP patients vs. 0% in the HP group (*p* < 0.0001). However, trans-esophageal echocardiographic (TEE) monitoring was more common in the SP group. Intraoperative hypotension was documented in 28% of SP patients compared to 9% in HP group (*p* = 0.0126). A higher proportion of SP patients experienced a new neurological symptom compared to the HP group (49 vs. 29%; *p* = 0.0281). The extent of tumor resection, postoperative 3-months survival, functional outcome, and patient satisfaction were not different in the groups.

**Conclusions:** The SP was associated with, less intraoperative bleeding, increased intraoperative hypotension, VAE, and postoperative neurological deficit. More HP patients experienced macroglossia and increased blood loss. At 3 months, there was no difference of parameters between the two groups.

**Clinical Trial Registration:** ClinicalTrials.gov: registration number NCT03364283.

## Introduction

Posterior cranial fossa (PCF) and pineal region (PR) surgery carries the risk of intraoperative damage to essential neurological structures and their supplying vasculature. Anatomical distortion may result from growing tumors in the posterior fossa, thus creating a serious challenge for the surgeon during tumor resection. Intraoperative monitoring and brainstem mapping can be applied to identify the normal structures within the brainstem and determine the safe margins of tumor resection (1–3).

One of the important factors while planning the surgery is patient positioning. An optimal position should be determined based on the intended surgical approach, location of the tumor or vascular malformation and its relationship with surrounding structures. Correct positioning of the patient will help to improve the intraoperative view, minimize the risk of mechanical trauma, retraction ischemia, and prevent development of significant brain edema (2, 4, 5).

Sitting position (SP) and horizontal position (HP) are used in PCF and PR surgeries, and the selection varies among surgical groups and countries (6). Both positions are relatively safe and useful but require team approach, experience and precautionary measures to minimize the risk of associated complications. In a group of 243 patients undergoing complex posterior fossa procedures, Spektor et al. found no significant associations between patients' position and surgical or neurological outcomes (7).

HP (prone, lateral, or park bench) is the preferred position in the majority of institutions due to simplicity and reduced risk of venous air embolism (VAE). However, it is not completely devoid of risks. Prone positioning may not ensure an optimal surgical view in some patients (8–13). The lateral position may result in patient instability on the operating table, increase the risk of brachial plexus injury, and predispose to muscular damage manifested by higher serum creatine kinase levels (8). Shimizu et al. describe a patient who developed a massive intraoperative neck swelling with subsequent brachial plexus injury during removal of a tentorial meningioma in park bench position (14).

HP has also been linked to development of airway obstruction, cervical cord compression, inferior vena cava compression, orbital compression, and retinal ischemia, postoperative macroglossia, necrosis at pressure points after prolonged surgery and other complications (10, 15, 16). There are numerous reports describing increased blood loss in HP when compared to SP (2, 7, 10, 17).

The advantages of SP include better anatomical orientation and surgical exposure, reduction in blood loss, better drainage of blood and cerebrospinal fluid from the operative field, easier access to the patient's face and chest by the anesthesiologist, and ease of cranial nerve monitoring (7, 9, 16).

VAE, airway edema, systemic arterial hypotension with decrease in cerebral perfusion pressure, central cord syndrome, jugular venous obstruction, pneumocephalus, paradoxical air embolism (PAE), and peripheral neuropathies are some of the reported complications related to the use of SP during PFS (18, 19). Despite the aforementioned risks, certain surgical and anesthetic advantages mentioned above make SP the preferred option for selected patients (2, 9, 12, 20–22). Historically, SP also allowed for intraoperative monitoring of spontaneous respiration in patients undergoing brainstem surgery. However, electrophysiological monitoring has largely replaced this practice (1, 23).

Both SP and HP have associated risks and potential benefits. While HP is preferred by majority of neurosurgeons because of reduced risk of VAE, there are conflicting data on neurological and functional outcome, success of surgery, patient satisfaction, overall morbidity, and mortality as well as intraoperative risks in groups operated on in HP and SP (10, 21).

This study was conducted to assess the effects of patient positioning on the rate of various intraoperative complications as well as functional recovery, morbidity, mortality, and the extent of surgery in patients undergoing PCF and PR surgery.

## Materials and Methods

The study was conducted in two medical centers: Ohio State University Wexner Medical Center (OSUMC), Columbus OH, USA, and Burdenko Neurosurgical Institute (BNI), Moscow, Russian Federation, between October 2015 and January 2017. The study was approved by The Ohio State University Institutional Review Board and by the Burdenko Neurosurgical Institute Ethics Committee. It was compliant with all institutional, state, and international regulations. Written informed consent was obtained from all the subjects before the surgical procedure. The trial is registered with www.ClinicalTrials.gov: registration number NCT03364283.

### Study Design

The study was designed as a prospective observational study conducted in two major medical centers treating significant numbers of neurosurgical patients. The data were prospectively collected in both institutions without intervening in medical decision-making. The patient positioning, type and extent of surgical intervention, and anesthesia management were the prerogatives of the medical teams in the corresponding institutions. In general, surgical and anesthetic managements were similar in both medical centers. There was a higher rate of intraoperative use of trans-esophageal echocardiographic (TEE) monitoring at the BNI site.

Patients were distributed into two major groups based on surgical position: SP and HP. All relevant intra- and postoperative data were analyzed for each institution to assess inter-institutional variability and as a pooled data set. All the surgeries were performed by neurosurgeons with significant experience in performing sitting craniotomies and using microsurgical technique. Postoperative neurological examinations were performed to compare with the preoperative level. The extent of tumor resection was assessed by routine early postoperative computerized tomographic and, in some patients, additional magnetic resonance imaging. The patients or their representatives were contacted by phone 3 months after surgery to assess the survival, functional recovery based on Glasgow Outcome Score (GOS), and patient satisfaction.

### Study Population

Patients with space-occupying or vascular lesions who were 18–75 years old and were scheduled for PCF or PR surgery were included in the study. The exclusion criteria were: history of significant cardio-vascular or chronic respiratory comorbid conditions, uncorrected hypovolemia, anemia, hypoalbuminemia; decompensated acid-base and electrolyte disorders; coagulation disorders, deep venous thrombosis; preexisting cervical myelopathy, cervical spine disorders preoperative evidence of spinal or peripheral nerve dysfunction that could interfere with patient positioning.

### Statistical Assessment

Demographic and clinical characteristics as well as surgical and post-operative outcomes were summarized for the two groups using descriptive statistics. Comparisons between SP and HP groups included clinical characteristics, surgical and post-operative complications and outcomes, as well as post-operative survival and patient satisfaction. Categorical variables were compared between groups using either a Chi-square test or Fisher's Exact Test, and continuous variables were compared with a two sample *t*-test or, when appropriate, Wilcoxon Rank Sum test. All data analyses were performed using SAS 9.4 (SAS Institute Inc., Cary, NC).

## Results

A total of 109 patients were included in the study for analysis. Overall, 53/109 patients (49%) were operated in SP and 56/109 patients (51%) in HP. At OSUMC, 38.46% of the surgical procedures were performed in SP and 61.54% in HP and at BNI, 42.71% were performed in SP and 57.29% in HP. There were no differences in demographics between the two study groups including age, weight, height, body mass index (BMI), gender, and race (Table 1). The clinical characteristics of the encountered surgical pathologies are summarized in Table 2.

**Table 1:** Demographic characteristics of the patients.

	**Sitting**	**Horizontal**	***P*-value**
	**(*n* = 53)**	**(*n* = 56)**	
Age (years), Median [IQR]	51 [37, 58]	48.5 [38, 57]	0.8390
Weight (kg), Median [IQR]	71.3 [59, 84]	68.3 [60, 82]	0.9492
Height (cm), Median [IQR]	1.7 [2, 1.7]	1.7 [2, 1.8]	0.9855
BMI (kg/m^2^), Median [IQR]	25.6 [22, 30.9]	25.9 [22, 29.3]	0.9275
Gender (Female)	37 (70%)	45 (80%)	0.2024
Race (Caucasian)	53 (100%)	54 (96%)	0.3813

*IQR, Inter-quartile Range*.

**Table 2:** Type and location of the surgical pathologies.

**Variable**	**Sitting (*n* = 53)**	**Horizontal (*n* = 56)**	***P*-value**
Chiari malformation type I	0 (0%)	7 (13%)	0.0130
Meningioma	21 (40%)	16 (29%)	0.2233
Neuroma	17 (32%)	32 (57%)	0.0085
Ependymoma	1 (2%)	1 (2%)	0.9687
Astrocytoma	2 (4%)	0 (0%)	0.2341
Cystic	3 (6%)	0 (0%)	0.1116
Metastatic	3 (6%)	0 (0%)	0.1116
Other tumors	4 (8%)	0 (0%)	0.0526
Vascular	2 (4%)	0 (0%)	0.2341
Cerebellar hemispheres	5 (9%)	0 (0%)	0.0246
Cerebellar vermis & rhomboid fossa	12 (23%)	9 (16%)	0.3847
Cranio-cervical location	2 (4%)	7 (13%)	0.1628
Cerebello-pontine angle	13 (25%)	29 (52%)	0.0056
Peripheral parenchyma	19 (36%)	11 (20%)	0.0583
Pineal region	2 (4%)	0 (0%)	0.2341

### Surgical Procedure

The median time required for surgical positioning was less for patients in the HP group compared to the SP group (5.5 min IQR [3, 7] vs. 10 min IQR [7, 15]). A higher proportion of patients in the HP group had >300 mL intraoperative blood loss compared to the SP group (18 [32%] vs. 7 [13%]; *p* = 0.0250). Episodes of intraoperative hypotension (<20% of initial values) were observed in 15 (28%) SP patients and in 5 (9%) HP patients, *p* = 0.0126. There were no differences observed between the two groups in duration of surgery, volume of intraoperative fluid administration, urinary output or the extent of tumor resection. The TEE was used for intraoperative monitoring in 40 (75%) patients undergoing surgery in SP and 2 (4%) in HP. The end-tidal CO_2_ (ETCO_2_) was monitored in all patients as part of the standard monitoring bundle. VAE was detected in 21 (40%) patients operated in SP. All patients with VAE were monitored with intraoperative TEE. None of the 56 patients in the HP group had intraoperative VAE (Table 3).

**Table 3:** Perioperative parameters.

**Variable**	**Sitting (*n* = 53)**	**Horizontal (*n* = 56)**	***P*-value**
Positioning time required (min), Median [IQR]	10 [7, 15]	5.5 [3, 7]	<0.0001
Anesthesia Duration (min), Median [IQR]	250 [220, 280]	245 [210, 302.5]	0.9589
Surgery Duration (min), Median [IQR]	180 [145, 210]	180 [145, 222.5]	0.8153
Blood loss volume (mL)			0.0250
<200 mL	18 (34%)	8 (14%)	
200 mL	11 (21%)	15 (27%)	
300 mL	17 (32%)	15 (27%)	
>300 mL	7 (13%)	18 (32%)	
Diuresis (mL), Median [IQR]	300 [200, 400]	300 [200, 500]	0.5740
Electrolyte infusion (mL), Median [IQR]	2,000 [1,600, 2,100]	1,600 [1,250, 1,850]	0.0704
Colloid infusion (mL), Median [IQR]	500 [500, 1,000]	500 [500, 500]	0.3709
Extubation time following surgery (min), Median [IQR]	90 [45, 127.5]	90 [55, 125]	0.8669
Extent of tumor resection (100 patients)			0.9175
Near gross total	8 (16%)^*^	8 (16%)^**^	
Subtotal	17 (33%)^*^	18 (37%)^**^	
Total	26 (51%)^*^	23 (47%)^**^	
Anesthesia complications	1 (2%)	1 (2%)	0.9687
Intraoperative hypertension	13 (25%)	15 (27%)	0.7875
Intraoperative hypotension	15 (28%)	5 (9%)	0.0126
VAE (N)	21 (40%)	0 (0%)	<0.0001
TEE	40 (75%)	2 (4%)	<0.0001
PETCO2	53 (100%)	56 (100%)	–
VAE 1 episode	14 (26%)	0 (0%)	<0.0001
VAE 2 episodes	6 (11%)	0 (0%)	0.0113
VAE 3 episodes	1 (2%)	0 (0%)	0.4862
PETCO2 baseline, Median [IQR]	38 [37, 40]	37 [36, 38]	0.0826

*IQR, Inter-quartile Range; VAE, Venous Air Embolism; TEE, Transesophageal Echocardiography*.

* *n = 51*.

** *n = 49*.

### Postoperative Complications

A greater proportion of patients in the SP group experienced new neurological symptoms compared to patients in the HP group (26 [49%] vs. 16 [29%], *p* = 0.0281). The postoperative dysfunction of the cranial nerves was not significantly different between the groups (24 [45%] vs. 16 [29%], *p* = 0.0704). Regarding other postoperative complications, macroglossia was observed in 2 (4%) patients in the HP group and in none in the SP group. Clinically significant postoperative pneumocephalus was present in 5 (9%) SP patients and in 6 (11%) HP patients (*p* = 0.8245) (Table 4).

**Table 4:** Postoperative complications.

**Variable**	**Sitting (*n* = 53)**	**Horizontal (*n* = 56)**	***P*-value**
Any neurological symptoms	26 (49%)	16 (29%)	0.0281
Abnormal sensory-motor function	2 (4%)	0 (0%)	0.2341
Cranial nerve function abnormal	24 (45%)	16 (29%)	0.0704
Peripheral neuropathy	2 (4%)	0 (0%)	0.2341
Spinal cord symptoms	0 (0%)	0 (0%)	–
Upper airway infection	1 (2%)	0 (0%)	0.4862
Macroglosia	0 (0%)	2 (4%)	0.4958
Pneumocephalus	5 (9%)	6 (11%)	0.8245

### Postoperative Outcome

Out of 53 patients in the SP group and 56 in the HP group, 14 (26%) and 6 (11%), respectively, were lost for follow-up. A patient in the SP group who underwent a total removal of the foramen magnum meningioma died as a result of postoperative complications not directly related to the intraoperative events. From the available data collected at 3 months, the survival rate was 38/39 patients (97%) in the SP group and 50/50 patients (100%) in the HP group. The functional outcome, GOS, and patient satisfaction data are presented in the Table 5.

**Table 5:** Postoperative outcome at 3 months.

**Variable**	**Sitting (*n* = 53)**	**Horizontal (*n* = 56)**	***P*-value**
Survival at 3 months	38 (72%)	50 (89%)	0.4382
Daily living help	6 (11%)	5 (9%)	0.5204
Daily assistance equipment	5 (9%)	3 (5%)	0.2837
Currently employed	13 (25%)	14 (25%)	0.5315
Able to return normal lifestyle	18 (34%)	28 (50%)	0.4971
GOS at 3 months			0.6145
GOS 3	0 (0%)	2 (4%)	
GOS 4	6 (11%)	7 (13%)	
GOS 5	32 (60%)	41 (73%)	
Patient Satisfaction/ Self-Assessment			0.3136
Improvement	21 (40%)	29 (52%)	
Same	9 (17%)	8 (14%)	
Worse	5 (9%)	13 (23%)	

*GOS, Glasgow Outcome Scale*.

## Discussion

The use of SP in neurosurgery has decreased in recent years due to its perceived association with serious complications and malpractice liability claims. The period of this decline coincides with successful litigation for neurological consequences after paradoxical air embolism (12). Ammirati et al. indicated that unproven assumptions of an increased rate of perioperative complications could be another cause of this fact (24). In a series of 1792 patients, Himes et al. found that the overall complication rate related to SP was 1.45% (1). The rate of VAE was 4.7%; however the rate of VAE requiring intervention was 1.06%. The authors conclude that when appropriately used with modern anesthesia techniques, the SP provides a safe means of surgical access. According to Hernesniemi et al., the SP is the preferred position in PR surgery. The authors performed PR surgeries in 119 patients, and only one patient was operated on in HP because of a preexisting cardiomyopathy. The authors encountered significant challenges with surgical approach and tumor resection in this patient that was related with HP (2).

HP for PCF and PR surgery, nevertheless, remains the preferred option for majority of medical centers. Israelyan et al. consider HP a safer option for patients undergoing microvascular decompression of the trigeminal nerve to treat neuralgia. The authors analyzed data of 200 patients undergoing trigeminal nerve decompression and found that while the risk of VAE is increased in SP craniotomy, the rate of postoperative liquorrhea is 4 times higher in surgery in HP. In their series, the neurological outcome was better in HP patients (13).

One of the principal concerns with SP is the development of VAE during surgery. There is a higher risk for development of VAE in SP, even though the HP is not completely devoid of that risk. Duke et al. undertook a retrospective review of 432 patients undergoing vestibular schwannoma surgeries is SP or HP. The authors found that despite the higher rate of intraoperative VAE in the SP group (28 vs. 5%) there was no significant difference in patient morbidity (25).

In order to minimize the risks of VAE in SP, Jadik et al. developed a standardized protocol, which includes preoperative TEE, intraoperative TEE monitoring, catheterization of the right atrium and a combination of fluid administration, positive end expiratory pressure, and standardized positioning aiming at a positive pressure in the transverse and sigmoid sinuses. Strict adherence to the established protocol and exclusion of patients with patent foramen ovale (PFO) helped to greatly minimize the risk of intraoperative VAE and its consequences (26). Likewise, in order to avoid thromboembolic events, mechanical (intermittent pneumatic compression), and pharmacological prophylaxis should be individually considered for patients both during and after the surgical intervention (27).

Thus, a significant variability in opinions and controversy exists in literature, and the majority of published studies are retrospective. Our study was designed as a prospective observational study in 2 major institutions treating complex neurosurgical patients and having common strategies in patient management.

Overall, 109 patients were included in the study. The most relevant intra- and postoperative data were recorded and analyzed (Tables 1–4). Besides the intraoperative parameters, patient satisfaction, neurological outcome at 3 months determined by GOS, and the extent of tumor resection were analyzed as well. The latter parameter was assessed based on the neuroimaging data before and after surgery.

In our study, all the VAE events were recorded in the SP group. In all cases, TEE was used for monitoring and allowed for detection of VAE in 21 SP patients (40%). Even though cerebral vessels may be a common location of air embolism in surgical and non-surgical patients (28), its reported incidence in patients undergoing cranial neurosurgical procedures is variable. Postoperative changes in patients' mental status may be a clinical sign of cerebral ischemia as a result of air embolism (29). However, there were no clinically significant findings associated with VAE in any of our patients.

Several techniques have been suggested to decrease the rate of VAE. Among them are: controlled hypoventilation, application of a neck tourniquet to increase the venous pressure, intravenous hydration therapy, application of venous compression on the legs (30). Preoperative screening is recommended to identify patients with PFO in an attempt to reduce the risk of intraoperative VAE in SP (26), even though paradoxical VAE may also be caused by transfer of air via the intrapulmonary shunts (31). TEE and precordial Doppler are considered the most sensitive methods for VAE detection. However, most of the VAE events are not associated with significant complications, and that correlates with our findings. Even though the TEE allows for rapid detection of VAE, no evidence of therapeutic benefit has been associated with its use (32). According to Gracia and Fabregas, the rate of false positive events will increase with TEE (6).

Other less sensitive methods used to detect VAE are precordial Doppler and monitoring of the ETCO_2_. Lossaso et al. found a detection threshold of 0.05 ml/kg of air using Doppler compared with the 0.15 ml/kg threshold for monitoring for PET CO_2_. Hemodynamic changes are the least sensitive and late indicators of VAE (33). Keeping a high index of suspicion for possible VAE and managing these cases promptly in order to limit their progression will significantly reduce the risks associated with SP (33).

The postoperative neurological outcome, patient satisfaction with the results of surgery, and the extent of tumor resection were analyzed as general indicators of successful treatment. Rath et al. reported that the lower cranial nerve function improved in 6.5% of patients operated on in the SP vs. 5.1% in the HP, preserved in 76.1% in the SP vs. 51.9% in the HP (*p* < 0.05), and deteriorated in 13.3% in the SP vs. 25.2% in the HP (*p* < 0.05) (16). In our patient groups, more patients in the SP group (49%) experienced new neurological symptoms compared to the HP group (29%) in the early postoperative period although the etiology of these new deficits is unclear and unlikely to be related to the surgical position. There was no difference observed in postoperative cranial nerve dysfunction between the groups, Table 4. The neurological outcome (determined by GOS) and patient satisfaction assessed at 3 months showed that there is no difference between groups.

We observed a higher proportion of patients in the HP group who experienced blood loss exceeding 300 mL compared to the SP group. However, the higher volume of blood loss was not clinically significant in our patients and did not require special correction. This may not be the case in pediatric population, and children may require a more precise correction of any intraoperative blood loss.

Recently, Gessler et al. reported an increased risk of cerebral venous and dural thrombosis (CVT) in patients undergoing cranial tumor removal in semi–sitting position. Prophylactic anticoagulation starting on postoperative day 1, postoperative ICU admission and postoperative imaging (including venous magnetic resonance angiography in patients with possible CVT) were some of the institutional practices described in this single-center retrospective study. The authors found an overall CVT incidence of 1.53% among 2,286 reviewed charts. A multivariate analysis identified age >55 years old, semi–sitting position (OR 7.55, 95% CI 3.73–15.31, *p* < 0.001) and other known comorbidities as the main risk factors for CVT (28, 29, 34).

The observational (non-interventional) design of our study had some limitations:

- The TEE was performed in a higher proportion of SP patients at BNI when compared with the OSUMC group, and thereby, more SP patients at BNI were diagnosed with VAE. However, those events were not clinically significant and did not affect the treatment outcome.
- The study did not reveal any significant differences among the patient groups in the neurological outcome based on the extent of tumor resection (partial vs. complete) or tumor location (e.g., pontocerebellar angle, pineal region, etc.). A larger multi-institutional study may help to reveal such differences, if any.
- An early postoperative computerized tomographic scan was performed as part of the standard clinical practice in our patient setting. Nevertheless, other imaging techniques such as postoperative magnetic resonance (e.g., to assess the incidence of cerebral venous air embolism and/or CVT) were not routinely used in all patients.

In conclusion, our study indicates that both SP and HP are relatively safe when used for PCF and PR surgery. The results are in concordance with the existing literature reports stating that both approaches may be safely used by experienced teams. Even though the SP is associated with significantly higher risk of VAE, major events requiring intervention are rare. In fact, the higher rate of VAE in the SP group did not affect the treatment outcome in our patients and was also related to a higher rate of intraoperative TTE monitoring in SP patient group. More patients in the SP group experienced neurological symptoms in the early postoperative period compared to the HP group. Nevertheless, the neurological recovery at 3 months was similar in both groups. No differences were observed in cranial nerve dysfunction, patient satisfaction, and the extent of tumor resection between the groups.

## Ethics Statement

The studies involving human participants were reviewed and approved by The Ohio State University. The patients/participants provided their written informed consent to participate in this study.

## Author Contributions

AM-M, LI, and JF-D subject recruitment, data collection, and manuscript writing. MP statistical analysis. SS, GS, VS, MA, AL, and SB manuscript writing and editing.

### Conflict of Interest

The authors declare that the research was conducted in the absence of any commercial or financial relationships that could be construed as a potential conflict of interest.

## References

[1] Himes BT Mallory GW Abcejo AS Pasternak J Atkinson JLD Meyer FB. Contemporary analysis of the intraoperative and perioperative complications of neurosurgical procedures performed in the sitting position. J Neurosurg. (2017) 127:182–8. 10.3171/2016.5.JNS152328 27494821

[2] Hernesniemi J Romani R Albayrak BS Lehto H Dashti R Ramsey C. Microsurgical management of pineal region lesions: personal experience with 119 patients. Surg Neurol. (2008) 70:576–83. 10.1016/j.surneu.2008.07.019 19055952

[3] Konig SA Spetzger U. Surgical strategies for supra– and infratentorially grown occipital meningeomas. J Neurol Surg A Cent Eur Neurosurg. (2012) 73:79–83. 10.1055/s-0032-1309061 22467480

[4] Silva JA Santos AA Jr Costa Mdo D Almeida EB. Suboccipital craniectomy with opening of the fourth ventricle and duraplasty: study of 192 cases of craniovertebral malformations. Arq Neuropsiquiatr. (2013) 71:609–14. 10.1590/0004-282X20130105 24141441

[5] Samii M Gerganov VM. Giant meningiomas of the posterior fossa. J Neurosurg. (2010) 112:905–6; discussion 6. 10.3171/2009.7.JNS091000 19877804

[6] Gracia I Fabregas N. Craniotomy in sitting position: anesthesiology management. Curr Opin Anaesthesiol. (2014) 27:474–83. 10.1097/ACO.0000000000000104 25051265

[7] Spektor S Fraifeld S Margolin E Saseedharan S Eimerl D Umansky F. Comparison of outcomes following complex posterior fossa surgery performed in the sitting versus lateral position. J Clin Neurosci. (2015) 22:705–12. 10.1016/j.jocn.2014.12.005 25752232

[8] Woernle CM Sarnthein J Foit NA Krayenbuhl N. Enhanced serum creatine kinase after neurosurgery in lateral position and intraoperative neurophysiological monitoring. Clin Neurol Neurosurg. (2013) 115:266–9. 10.1016/j.clineuro.2012.05.011 22682772

[9] Orliaguet GA Hanafi M Meyer PG Blanot S Jarreau MM Bresson D Is the sitting or the prone position best for surgery for posterior fossa tumours in children? Pediatr Anesth. (2001) 11:541–7. 10.1046/j.1460-9592.2001.00733.x 11696117

[10] Black S Ockert DB Oliver JW Cucchiara RF. Outcome following posterior fossa craniectomy in patients in the sitting or horizontal positions. Anesthesiology. (1988) 69:49–56. 10.1097/00000542-198807000-00008 3389566

[11] Charbel F Kehrli P Pain L editors. La position Assise en Neurochirurgie: le Point de vue du chirurgien. Annales francaises d'anesthesie et de Reanimation. Masson: Elservier Masson (1998). 10.1016/S0750-7658(98)80067-1 9750715

[12] Porter JM Pidgeon C Cunningham AJ. The sitting position in neurosurgery: a critical appraisal. Br J Anaesth. (1999) 82:117–28. 10.1093/bja/82.1.117 10325848

[13] Israelian L Shimanskii V Otamanov D Poshataev V Lubnin A Patient positioning on the operating table in neurosurgery: sitting or lying. Anesteziol Reanimatol. (2013) 4:18–26. 24341037

[14] Shimizu S Sato K Mabuchi I Utsuki S Oka H Kan S. Brachial plexopathy due to massive swelling of the neck associated with craniotomy in the park bench position. Surg Neurol. (2009) 71:504–8. 10.1016/j.surneu.2007.08.043 18207495

[15] Rau CS Liang CL Lui CC Lee TC Lu K. Quadriplegia in a patient who underwent posterior fossa surgery in the prone position. case report. J Neurosurg. (2002) 96 (Suppl. 1):101–3. 10.3171/spi.2002.96.1.0101 11795695

[16] Rath GP Bithal PK Chaturvedi A Dash HH. Complications related to positioning in posterior fossa craniectomy. J Clin Neurosci. (2007) 14:520–5. 10.1016/j.jocn.2006.02.010 17430775

[17] Harrison EA Mackersie A McEwan A Facer E. The sitting position for neurosurgery in children: a review of 16 years' experience. Br J Anaesth. (2002) 88:12–7. 10.1093/bja/88.1.12 11881865

[18] Fathi AR Eshtehardi P Meier B. Patent foramen ovale and neurosurgery in sitting position: a systematic review. Br J Anaesth. (2009) 102:588–96. 10.1093/bja/aep063 19346525

[19] Gale T Leslie K. Anaesthesia for neurosurgery in the sitting position. J Clin Neurosci. (2004) 11:693–6. 10.1016/j.jocn.2004.05.007 15337126

[20] Bruce JN. Sitting position for the removal of pineal region lesions. World Neurosurg. (2012) 77:657–8. 10.1016/j.wneu.2011.03.015 22079819

[21] Sanai N Mirzadeh Z Lawton MT. Supracerebellar–supratrochlear and infratentorial–infratrochlear approaches: gravity–dependent variations of the lateral approach over the cerebellum. Oper Neurosurg. (2010) 66 (Suppl. 2):264–74. 10.1227/01.NEU.0000369653.12185.FD 20489515

[22] Giannini A Bricchi M. Posterior fossa surgery in the sitting position in a pregnant patient with cerebellopontine angle meningioma. Br J Anaesth. (1999) 82:941–4. 10.1093/bja/82.6.941 10562796

[23] Gunther F Frank P Nakamura M Hermann EJ Palmaers T. Venous air embolism in the sitting position in cranial neurosurgery: incidence and severity according to the used monitoring. Acta Neurochir. (2017) 159:339–46. 10.1007/s00701-016-3034-7 27896454

[24] Ammirati M Lamki TT Shaw AB Forde B Nakano I Mani M. A streamlined protocol for the use of the semi–sitting position in neurosurgery: a report on 48 consecutive procedures. J Clin Neurosci. (2013) 20:32–4. 10.1016/j.jocn.2012.05.037 23178073 PMC3840951

[25] Duke DA Lynch JJ Harner SG Faust RJ Ebersold MJ. Venous air embolism in sitting and supine patients undergoing vestibular schwannoma resection. Neurosurgery. (1998) 42:1282–6; discussion 6–7. 10.1097/00006123-199806000-00047 9632186

[26] Jadik S Wissing H Friedrich K Beck J Seifert V Raabe A. A standardized protocol for the prevention of clinically relevant venous air embolism during neurosurgical interventions in the semisitting position. Neurosurgery. (2009) 64:533–8; discussion 8–9. 10.1227/01.NEU.0000338432.55235.D3 19240616

[27] Ganau M Prisco L Cebula H Todeschi J Abid H Ligarotti G. Risk of deep vein thrombosis in neurosurgery: state of the art on prophylaxis protocols and best clinical practices. J Clin Neurosci. (2017) 45:60–6. 10.1016/j.jocn.2017.08.008 28890040

[28] McCarthy CJ Behravesh S Naidu SG Oklu R. Air embolism: diagnosis, clinical management and outcomes. Diagnostics. (2017) 7:5. 10.3390/diagnostics7010005 28106717 PMC5373014

[29] Mirski MA Lele AV Fitzsimmons L Toung TJ. Diagnosis and treatment of vascular air embolism. Anesthesiol J Am Soc Anesthesiol. (2007) 106:164–77. 10.1097/00000542-200701000-00026 17197859

[30] Lubnin A Oskanova M. [Prevention of air embolism in neurosurgical patients operated on in a sitting posture: a comparative study of 3 methods]. Anesteziol Reanimatol. (1994) 22–6. 7893069

[31] Anan'ev EP Polupan AA Savin IA Goryachev AS Troitskiy AP Kolokol'nikov AE. [Paradoxical air embolism resulted in acute myocardial infarction and massive ischemic brain injury in a patient operated on in a sitting position]. Zh Vopr Neirokhir Im N N Burdenko. (2016) 80:84–92. 10.17116/neiro201680284-92 27070262

[32] Wong AY Irwin MG. Large venous air embolism in the sitting position despite monitoring with transoesophageal echocardiography. Anaesthesia. (2005) 60:811–3. 10.1111/j.1365-2044.2005.04237.x 16029232

[33] Losasso TJ Muzzi DA Dietz NM Cucchiara RF. Fifty percent nitrous oxide does not increase the risk of venous air embolism in neurosurgical patients operated upon in the sitting position. Anesthesiology. (1992) 77:21–30. 10.1097/00000542-199207000-00005 1609997

[34] Gessler F Bruder M Duetzmann S Tritt S Bernstock JD Seifert V. Risk factors governing the development of cerebral vein and dural sinus thrombosis after craniotomy in patients with intracranial tumors. J Neurosur. (2017) 128:373–9. 10.3171/2016.11.JNS161871 28387630

